# Analytical and Workflow Performance of a High‐Throughput Chemiluminescent Immunoassay System in a Clinical Laboratory

**DOI:** 10.1002/jcla.70270

**Published:** 2026-05-27

**Authors:** Chiung‐Tzu Hsiao, Chao‐Ching Liao, Yu‐Chang Chang, Hui‐Ju Chang, Tz‐Ya Huang, Jen‐Shiou Lin, Po‐Ren Hsueh

**Affiliations:** ^1^ Department of Laboratory Medicine China Medical University Hospital, China Medical University Taichung City Taiwan; ^2^ Department of Laboratory Medicine, Taichung Municipal Geriatric Rehabilitation General Hospital China Medical University Taichung City Taiwan

**Keywords:** analytical performance, chemiluminescent immunoassay, DxI 9000 Access Immunoassay System, method comparison, reference interval verification

## Abstract

**Background:**

Increasing test volumes and shorter turnaround time (TAT) requirements, particularly in acute‐care settings, challenge clinical laboratories to maintain accurate immunoassay testing. High‐throughput chemiluminescent immunoassay systems must therefore provide robust analytical performance and efficient workflow integration. This study evaluated the analytical performance and selected workflow characteristics of one such system under routine clinical laboratory conditions in Taiwan.

**Methods:**

Thirty‐six analytes were evaluated at a tertiary‐care medical center. Analytical performance assessments included precision, accuracy, limit of blank (LoB), method comparison, carryover, and reference interval verification, conducted in accordance with relevant Clinical and Laboratory Standards Institute (CLSI) guidelines. Selected workflow characteristics, including pipettor consistency, low‐volume precision, and time‐to‐first‐result (TTFR) for high‐sensitivity troponin I (hsTnI), beta‐human chorionic gonadotropin (β‐hCG), and procalcitonin (PCT)—were also assessed to evaluate operational suitability for STAT testing.

**Results:**

All analytes demonstrated stable performance, with within‐run coefficients of variation (CV) generally below 4% and within‐laboratory CV predominantly below 5%. Accuracy verification met peer‐group or external quality assessment acceptance criteria for all assays. LoB values met or exceeded manufacturer specifications. Method comparison showed excellent agreement with established platforms (coefficients of determination [*R* ^2^] > 0.95; bias within 0.8 × total allowable error [TEa]). Carryover was negligible, reference interval verification met acceptance criteria, and TTFR for STAT assays was reduced by at least 5 min compared with the predecessor platform.

**Conclusion:**

This evaluation demonstrated that the assessed high‐throughput chemiluminescent immunoassay system provided reliable analytical performance across a broad test menu and improved STAT turnaround time, supporting its routine use in hospital laboratories.

## Graphical abstract

Analytical and operational performance evaluation of the DxI 9000 Access Immunoassay Analyzer in a clinical laboratory in Taiwan.

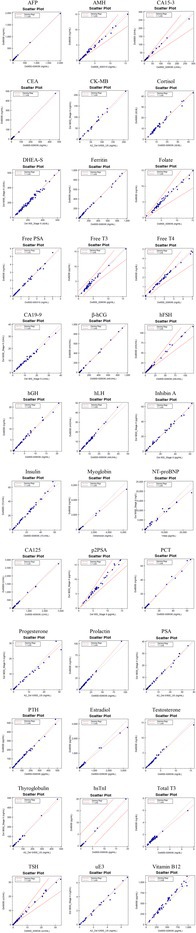

## 1 Introduction

Hospital‐based clinical laboratories are facing increasing demands driven by rising test volumes, shorter TAT expectations, and progressively stringent accreditation and regulatory requirements [1, 2]. In parallel, the implementation of updated Clinical Laboratory Improvement Amendments (CLIA) 2024 total allowable error standards has further underscored the need for consistent analytical precision and reliable measurement performance in routine clinical practice [3]. Within this context, immunoassay platforms must not only deliver analytically robust results but also support efficient laboratory workflows capable of meeting acute‐care and high‐throughput testing demands.

To address these combined analytical and operational requirements, high‐throughput chemiluminescent immunoassay systems have been developed with the aim of improving assay precision, processing efficiency, and workflow integration while maintaining compatibility with established assay menus. The DxI 9000 Access Immunoassay Analyzer (Beckman Coulter, Brea, CA, USA) represents one such platform designed to support routine clinical laboratory operation under conditions of increasing workload and regulatory scrutiny.

Previous evaluations conducted across multiple sites have reported that assays performed on the DxI 9000 achieve analytical performance consistent with Six Sigma quality targets and meet CLIA 2024 performance requirements across diverse assay categories [3, 4, 5]. In addition, published study focusing on clinically relevant infectious disease assays, including hepatitis B surface antigen and anti–hepatitis B core total, have demonstrated reliable analytical performance of the DxI 9000 under routine diagnostic conditions [4, 6]. These findings are aligned with broader reports indicating that contemporary chemiluminescent immunoassay systems provide improved analytical consistency compared with legacy platforms, particularly for viral hepatitis B and human immunodeficiency virus testing [2, 7].

Collectively, these studies indicate that modern high‐throughput immunoassay platforms can achieve both analytical robustness and compliance with evolving regulatory requirements. However, most published evaluations have focused on selected assay groups or specific clinical applications, leaving limited data on integrated analytical performance across a broad test menu within a single routine laboratory environment.

To address this gap, the present study evaluated 36 immunoassay analytes selected to reflect routine diagnostic demands across major clinical service lines. The analyte set encompasses assays with diverse antigen–antibody characteristics and matrix complexities, enabling assessment of analytical performance across a wide range of analyte concentrations and varied clinical contexts.

By integrating comprehensive analytical performance verification with selected workflow‐related assessments, this study aimed to characterize the suitability of a high‐throughput chemiluminescent immunoassay system for routine use in a tertiary‐care hospital laboratory. Accordingly, a structured analytical performance evaluation was conducted under routine clinical laboratory conditions at a university‐affiliated tertiary medical center in Taiwan, following established methodological guidelines [1, 8].

## 2 Materials and Methods

### 2.1 Instrumentation and Study Design

This analytical performance evaluation was conducted at China Medical University Hospital (CMUH), a 2200‐bed university‐affiliated tertiary‐care medical center in Taiwan, in accordance with CMUH's institutional Test Development and Assessment Management Procedure, with internal review and approval completed prior to study initiation. The DxI 9000 Access Immunoassay Analyzer (Beckman Coulter, Brea, CA, USA) was installed, calibrated, and verified for operational readiness by certified service engineers before analytical testing, and all recommended preventive maintenance procedures were completed according to the manufacturer's instructions.

All evaluations were performed in accordance with applicable CLSI guidelines, including precision evaluation (EP05‐A3), user performance verification (EP15‐A2), method comparison and bias estimation (EP09‐A3), detection capability assessment (EP17‐A2), and reference interval verification (EP28‐A3c) [9, 10, 11, 12, 13]. Carryover assessment followed EP Evaluator carryover interpretation guidelines [14]. Statistical analyses were performed using EP Evaluator software (EE12, licensed version; Beckman Coulter, Brea, CA, USA) [15].

Thirty‐six immunoassay analytes across seven diagnostic domains—tumor markers, thyroid function, reproductive hormones, cardiac biomarkers, bone and mineral metabolism, growth and stress‐related hormones, and metabolic and inflammatory markers—were evaluated.

Clinical specimens consisted of lithium heparin plasma or serum samples obtained from routine clinical testing. Lithium heparin plasma samples were collected using BD Vacutainer LH (Lithium Heparin) PST II Plus blood collection tubes (Cat. No. 367376) for CK‐MB, NT‐proBNP, hsTnI, β‐hCG, and PCT assays, whereas serum samples were collected using BD Vacutainer Serum Separator Tubes (SST; Cat. No. 367986) for all remaining analytes.

Following collection, samples were processed through the automated laboratory track system and centrifuged at 3000 rpm for 5 min. Residual clinical specimens were aliquoted into polypropylene tubes and stored at −20°C until analysis. Before testing, samples were thawed once at room temperature, mixed thoroughly, and centrifuged again at 3000 rpm for 5 min prior to loading onto the analyzer.

All assays were performed using commercially available reagent kits, calibrators, and quality control materials validated for use on the DxI 9000 platform. Quality control materials included Liquichek Tumor Marker Control (Lot 94,960), Liquichek Cardiac Markers Plus Control (Lot 67,700), Liquichek Immunoassay Plus Control (Lot 85,340), Liquichek Specialty Immunoassay Control (Lot 65,000), Lyphochek Specialty Immunoassay Control (Lot 88,730), Access AMH QC (Lot 389,208), Access Inhibin A QC (Lot 440,393), and Access p2PSA QC Control Kit (Lot 440,355). Reagents and control materials were stored at 2°C–8°C, and specimen handling and testing complied with internationally recognized ethical principles governing the secondary use of human biological materials [16].

### 2.2 Precision

Precision was evaluated in accordance with the CLSI EP05‐A3 guideline [9]. Repeatability (within‐run precision) was assessed using two levels of quality control (QC) materials representing low‐ and high‐concentration ranges (QC1 and QC3). Each QC level was measured 20 consecutive times within a single analytical run.

Within‐laboratory imprecision was assessed using the same QC materials. For each analyte, the two QC levels were analyzed four times per day over five consecutive days, resulting in 20 measurements per concentration level.

Quality control materials consisted of third‐party controls from Bio‐Rad Laboratories (Hercules, CA, USA) and manufacturer‐provided controls from Beckman Coulter. QC materials were stored at 2°C–8°C according to manufacturer instructions and equilibrated to room temperature prior to analysis.

Mean values, standard deviations (SD), and CV were calculated using EP Evaluator software (EE12; Beckman Coulter, Brea, CA, USA), and results were evaluated against manufacturer‐defined acceptance criteria, consistent with established verification practices for quantitative immunoassays [17].

### 2.3 Accuracy

Accuracy was evaluated by comparing results obtained on the DxI 9000 with peer‐group target ranges or assigned values from external quality assessment (EQA) programs, where available. For each analyte, two to three QC concentration levels were tested in duplicate, with residual EQA specimens included when available. Single EQA measurements were accepted provided that at least two QC concentration levels were assessed concurrently.

For analytes lacking established peer‐group target values, including unconjugated estriol (uE3), [−2] pro‐prostate‐specific antigen (p2PSA), and inhibin A, EQA‐assigned target intervals were used as reference standards. When interlaboratory participation was limited (< 10 participating sites) and SD values were unavailable, SD estimates derived from analytically equivalent Access assays at comparable concentration levels were used to define ±3 SD acceptance limits. For N‐terminal pro‐B‐type natriuretic peptide (NT‐proBNP), manufacturer‐provided reference limits were applied in the absence of peer‐group or EQA‐assigned targets.

Analytical acceptability was defined as results falling within the applicable peer‐group, EQA‐assigned, or manufacturer‐specified acceptance ranges. This evaluation strategy is consistent with previously reported accuracy assessment approaches for integrated immunoassay systems [1].

### 2.4 LoB

Analytical sensitivity was assessed by determining the LoB in accordance with the CLSI EP17‐A2 guideline [12]. Matrix blank samples, consisting of assay diluents or zero calibrators (S₀), were analyzed in at least 10 replicate measurements for each analyte. The LoB was calculated using the EP17‐A2–recommended equation (LoB = μ_blank + 1.645 × σ_blank), corresponding to the one‐sided 95th percentile of the blank distribution. Statistical analyses were performed using EP Evaluator software (EE12; Beckman Coulter, Brea, CA, USA). LoB performance was considered acceptable when calculated values met or were lower than the corresponding manufacturer performance claims. This evaluation approach is consistent with previously reported analytical sensitivity assessments for clinical immunoassays [18, 19, 20].

### 2.5 Method Comparison

Method comparison studies were performed between the DxI 9000 Access Immunoassay Analyzer (Beckman Coulter, Brea, CA, USA) and established analytical platforms routinely used in the clinical laboratory. The comparator systems included the UniCel DxI 800 Immunoassay System (Beckman Coulter, Brea, CA, USA), which employs chemiluminescent immunoassay technology, the Dimension EXL 200 (Dimension) Integrated Chemistry System (Siemens Healthineers, Erlangen, Germany) based on LOCI (Luminescent Oxygen Channeling Immunoassay) advanced chemiluminescence technology, and the VIDAS 3 system (bioMérieux, Marcy‐l'Étoile, France), which utilizes enzyme‐linked fluorescent assay (ELFA) technology.

Residual patient specimens obtained during routine clinical testing were used for comparison analysis. Specimens were selected to represent the widest possible analytical measurement range encountered in routine clinical practice. To minimize potential preanalytical variability, paired measurements on the DxI 9000 and comparator platforms were performed within the same analytical timeframe whenever feasible.

For most analytes, approximately 40 patient specimens were included, consistent with the general recommendations of the CLSI EP09‐A3 guideline for method comparison studies.

However, for several assays—CK‐MB, progesterone, thyroglobulin, unconjugated estriol (uE3), [−2] pro‐prostate‐specific antigen (p2PSA), N‐terminal pro‐B‐type natriuretic peptide (NT‐proBNP), and CA 125—the number of available specimens was below 40 because of limited specimen availability and relatively lower routine testing frequency during the defined study period. For these analytes, all eligible specimens meeting the inclusion criteria were included in the analysis to maximize representation of clinically relevant concentration ranges.

Statistical evaluation was conducted using EE12 software (Beckman Coulter), applying Deming regression and bias estimation as appropriate. Analytical comparability was considered acceptable when the R^2^ exceeded 0.95 and the observed bias remained within 0.8 × TEa, reflecting a balance between statistical correlation and clinically meaningful agreement. These acceptance criteria are consistent with established method comparison practices and published recommendations for immunoassay validation [18, 21].

### 2.6 Carryover

Carryover was evaluated for β‐hCG and hsTnI using an alternating sequence of low‐ and high‐concentration samples (L1–L11 and H1–H10). For statistical assessment, the mean and SD of predefined low‐level replicates (L2, L3, L6, L7, L8) were compared with those of high‐to‐low transition replicates (L4, L5, L9, L10, L11). Carryover was calculated as the difference between the mean values of the High–Low and Low–Low sample sets. Acceptability was defined as carryover not exceeding three times the SD of the Low–Low replicates. All calculations were performed using EE12 statistical software, and result interpretation followed established carryover evaluation guidelines [14].

### 2.7 Reference Interval Verification

Reference interval verification was performed in accordance with the CLSI EP28‐A3c guideline [13]. For each applicable analyte, 20 specimens from apparently healthy individuals were analyzed, and verification was considered acceptable when at least 18 of 20 results (≥ 90%) fell within the corresponding manufacturer‐defined reference interval.

Due to known sex‐related physiological variability, only male specimens were used for verification of estradiol and prolactin, while female reference intervals were adopted from manufacturer documentation or established tertiary‐care reference sources. For NT‐proBNP, verification was restricted to individuals younger than 75 years to account for age‐dependent biomarker distribution. Assays without established manufacturer‐defined reference intervals, as well as analytes based on derived calculations or clinical decision thresholds rather than population‐based reference limits—including p2PSA and free prostate‐specific antigen (Free PSA)—were excluded from verification, consistent with the scope of reference interval verification defined by the EP28‐A3c guideline.

### 2.8 Additional Functional Evaluations

In addition to core analytical assessments, three supplemental evaluations were conducted to further characterize functional performance attributes of the DxI 9000 platform. These included assessment of pipettor consistency, low‐volume precision, and TTFR.

### 2.9 Pipettor Consistency

Inter‐pipettor variability was evaluated using estradiol, hsTnI, and thyroid‐stimulating hormone (TSH). Three control levels were tested across all four pipettors, with 12–15 replicate measurements per level. Percent bias among pipettors was calculated to assess consistency of sample dispensing performance.

### 2.10 Low‐Volume Precision

To simulate testing under limited sample‐volume conditions, alpha‐fetoprotein (AFP; 4 μL) and β‐hCG (2 μL) were analyzed in dilution mode. CV were calculated to evaluate analytical stability under minimal sample input conditions.

### 2.11 TTFR

Using the DxI 9000's upgraded chemiluminescent substrate (Lumi‐phos Pro), TTFR was assessed with sequential STAT patient samples for hsTnI, β‐hCG, and PCT. For each assay, 13–16 consecutive specimens were processed, and the elapsed time from sample loading to result availability was recorded.

## 3 Results

### 3.1 Precision

Precision results are summarized in Table 1. Across the 36 analytes evaluated, within‐run CV for both low‐ and high‐level quality control materials was generally below 4%. Although total triiodothyronine (Total T3) demonstrated relatively higher variability, all analytes met the manufacturer‐defined precision acceptance criteria.

**TABLE 1:** Within‐run and within‐laboratory precision results for 36 analytes measured on the DxI 9000 Access Immunoassay System, expressed as mean concentrations and CV.

Analyte	Unit	Within‐run precision		Within‐laboratory precision	
		Mean (QC1/QC3)	CV (%) (QC1/QC3)	Mean (QC1/QC3)	CV (%) (QC1/QC3)
AFP	ng/mL	7.920/203.986	3.4/2.6	8.630/231.090	3.3/3.1
AMH	ng/mL	1.052/15.572	1.8/1.0	0.981/14.837	4.8/3.3
CA15‐3	U/mL	8.92/53.62	1.7/1.6	9.93/57.16	3.1/3.5
CEA	ng/mL	2.272/70.037	2.9/3.0	2.556/75.164	3.6/3.4
CK‐MB	ng/mL	2.95/66.78	2.2/2.3	3.00/68.74	3.2/4.3
Cortisol	μg/dL	4.191/34.011	3.6/2.8	3.662/30.709	5.1/3.6
DHEA‐S	μg/dL	81.82/505.13	2.7/2.5	83.28/505.53	3.9/4.2
Ferritin	ng/mL	21.23/343.54	2.0/3.5	21.68/377.75	2.8/4.2
Folate	ng/mL	2.678/11.741	2.2/0.9	2.764/11.849	4.8/3.5
Free PSA	ng/mL	0.328/12.75	2.6/1.9	0.320/11.987	4.1/3.0
Free T3	pg/mL	2.688/10.382	2.4/2.1	2.471/10.627	2.8/2.3
Free T4	ng/dL	0.726/3.877	2.5/2.4	0.747/3.817	2.4/2.5
CA19‐9	U/mL	20.04/287.76	2.0/1.5	19.71/285.11	2.2/1.8
β‐hCG	mIU/mL	6.158/397.596	1.9/1.6	6.067/399.192	1.9/1.8
hFSH	mIU/mL	9.076/41.845	2.1/2.9	9.089/42.387	3.1/3.6
hGH	ng/mL	4.245/18.229	2.1/2.2	4.474/19.532	1.8/3.2
hLH	mIU/mL	4.837/59.713	1.7/2.0	4.852/60.837	2.5/2.0
Inhibin A	pg/mL	155.58/786.57	2.5/2.1	151.97/792.39	3.1/2.3
Insulin	μIU/mL	16.801/178.781	1.9/1.9	17.223/170.119	2.7/2.3
Myoglobin	ng/mL	52.87/208.4	2.3/2.1	43.77/226.47	3.7/3.1
NT‐proBNP	ng/L	160/6120.9	3.3/2.4	139.3/5210.3	3.1/1.9
CA125	U/mL	47.53/341.9	1.7/2.2	49.03/356.5	5.1/1.2
p2PSA	pg/mL	23.729/1077.801	2.2/0.9	25.088/1116.323	3.5/2.0
PCT	ng/mL	1.009/31.321	2.7/1.9	0.794/25.468	4.4/4.7
Progesterone	ng/mL	0.881/19.103	2.8/2.3	0.881/19.691	4.5/3.6
Prolactin	ng/mL	7.245/41.808	1.3/1.8	8.169/45.946	2.9/1.8
PSA	ng/mL	0.171/19.883	2.6/1.5	0.178/20.618	3.1/2.4
PTH	pg/mL	31.96/702.22	1.9/1.5	33.00/753.68	2.2/1.9
Estradiol	pg/mL	65.760/710.687	2.9/3.3	61.948/722.583	4.8/1.1
Testosterone	ng/dL	1.116/7.445	3.4/2.1	0.963/7.370	4.7/2.1
Thyroglobulin	ng/mL	4.367/5.716	2.0/2.5	4.290/5.725	2.9/2.9
hsTnI	pg/mL	0.013/4.907	3.2/1.6	0.013/4.650	3.0/2.3
Total T3^a^	ng/dL	100.9/343.4	4.0/2.7	108.1/357.0	5.3/3.9
TSH	μIU/mL	0.798/37.866	3.4/2.9	0.778/38.155	4.6/4.5
uE3	ng/mL	2.762/6.217	2.0/2.6	2.717/6.232	4.7/3.9
Vitamin B12	pg/mL	175.2/551.3	2.9/2.4	181.1/586.0	5.0/2.8

Abbreviations: AFP, alpha‐fetoprotein; AMH, anti‐Müllerian hormone; CA125, cancer antigen 125; CA15‐3, cancer antigen 15‐3; CA19‐9, cancer antigen 19‐9; CEA, carcinoembryonic antigen; CK‐MB, creatine kinase‐MB; CV, coefficient of variation; DHEA‐S, dehydroepiandrosterone sulfate; Free PSA, free prostate‐specific antigen; Free T3, free triiodothyronine; Free T4, free thyroxine; hFSH, human follicle‐stimulating hormone; hGH, human growth hormone; hLH, human luteinizing hormone; hsTnI, high‐sensitivity troponin I; NT‐proBNP, N‐terminal pro‐B‐type natriuretic peptide; p2PSA, [−2] pro‐prostate‐specific antigen; PCT, procalcitonin; PSA, prostate‐specific antigen; PTH, parathyroid hormone; QC, quality control; Total T3, total triiodothyronine; TSH, thyroid‐stimulating hormone; uE3, unconjugated estriol; β‐hCG, beta‐human chorionic gonadotropin.

^a^ Total T3 showed comparatively higher variability in precision testing but remained within manufacturer‐defined acceptability criteria.

Within‐laboratory precision assessed over five consecutive days showed CV values predominantly below 5%, with all results remaining within the predefined acceptance limits.

### 3.2 Accuracy

Accuracy results are summarized in Table 2. All evaluated analytes met their respective acceptance criteria when compared with peer‐group target ranges or assigned values from external quality assessment programs. Results across the assessed test menu, including tumor markers, reproductive and adrenal hormones, cardiac biomarkers, thyroid function assays, and vitamins, were within the predefined acceptance limits.

**TABLE 2:** Accuracy evaluation for 36 analytes on the DxI 9000 Access Immunoassay System.

Analyte	Unit	Test range	Levels (*n*)
AFP	ng/mL	6.75–180.40	3
AMH	ng/mL	0.99–15.06	3
CA 15‐3	U/mL	9.1–55.7	3
CEA	ng/mL	2.18–64.88	2
CK‐MB	ng/mL	3.0–66.5	3
Cortisol	μg/dL	4.23–32.71	3
DHEA‐S	μg/dL	77.8–453.3	3
Ferritin	ng/mL	21.4–349.3	3
Folate	ng/mL	3.17–13.27	3
Free PSA	ng/mL	0.34–12.25	3
Free T3	pg/mL	2.45–10.15	3
Free T4	ng/dL	0.79–3.80	3
CA19‐9	U/mL	19.9–288.5	3
β‐hCG	mIU/mL	5.87–383.90	3
hFSH	mIU/mL	8.70–40.73	3
hGH	ng/mL	4.23–17.90	3
hLH	mIU/mL	4.76–58.84	3
Inhibin A	pg/mL	44.6–243.8	3
Insulin	μIU/mL	16.55–157.30	3
Myoglobin	ng/mL	50.6–208.4	5
NT‐proBNP	ng/L	153–5643	3
CA125	U/mL	48.5–363.2	3
p2PSA	pg/mL	0.26–3.48	3
PCT	ng/mL	0.76–24.51	3
Progesterone	ng/mL	1.08–23.75	5
Prolactin	ng/mL	7.77–42.45	3
PSA	ng/mL	0.17–17.98	2
PTH	pg/mL	30.4–712.3	3
Estradiol	pg/mL	64.61–726.50	3
Testosterone	ng/dL	1.08–7.53	3
Thyroglobulin	ng/mL	4.14–106.70	3
hsTnI	pg/mL	14.0–5120.0	3
Total T3	ng/dL	97–334	3
TSH	μIU/mL	0.751–35.520	3
uE3	ng/mL	0.096–3.200	8
Vitamin B12	pg/mL	176–566	3

*Note:* Accuracy was confirmed across all analytes, with results meeting acceptance criteria based on peer‐group, external quality assessment, or manufacturer reference targets.

Abbreviations: AFP, alpha‐fetoprotein; AMH, anti‐Müllerian hormone; CA 125, cancer antigen 125; CA 15‐3, cancer antigen 15‐3; CA 19‐9, cancer antigen 19‐9; CEA, carcinoembryonic antigen; CK‐MB, creatine kinase‐MB; DHEA‐S, dehydroepiandrosterone sulfate; Free PSA, free prostate‐specific antigen; Free T3, free triiodothyronine; Free T4, free thyroxine; hFSH, human follicle‐stimulating hormone; hGH, human growth hormone; hLH, human luteinizing hormone; hsTnI, high‐sensitivity troponin I; NT‐proBNP, N‐terminal pro‐B‐type natriuretic peptide; p2PSA, [−2] pro‐prostate‐specific antigen; PCT, procalcitonin; PSA, prostate‐specific antigen; PTH, parathyroid hormone; Total T3, total triiodothyronine; TSH, thyroid‐stimulating hormone; uE3, unconjugated estriol; β‐hCG, beta‐human chorionic gonadotropin.

### 3.3 LoB

LoB results are summarized in Table 3. For all evaluated analytes, observed LoB values met or were lower than the corresponding manufacturer‐stated performance claims. Several assays exhibited LoB values substantially lower than the manufacturer specifications, including AFP (0.02 ng/mL vs. 0.5 ng/mL), TSH (0.0000 μIU/mL vs. 0.005 μIU/mL), ferritin (0.13 ng/mL vs. 0.2 ng/mL), β‐hCG (0.021 mIU/mL vs. 0.6 mIU/mL), and vitamin B12 (8.9 pg/mL vs. 50 pg/mL).

**TABLE 3:** LoB evaluation results for 36 analytes on the DxI 9000 Access Immunoassay System, compared with manufacturer specifications.

Analyte	Unit	LoB	Manufacturer claim
AFP	ng/mL	0.02	0.5
AMH	ng/mL	0.000	0.02
CA15‐3	U/mL	0.00	0.5
CEA	ng/mL	0.008	0.1
CK‐MB	ng/mL	0.00	0.1
Cortisol	μg/dL	0.000	0.4
DHEA‐S	μg/dL	0.48	2
Ferritin	ng/mL	0.13	0.2
Folate	ng/mL	0.000	1
Free PSA	ng/mL	0.0000	0.005
Free T3	pg/mL	0.259	0.88
Free T4	ng/dL	0.098	0.25
CA19‐9	U/mL	0.00	0.8
β‐hCG	mIU/mL	0.021	0.6
hFSH	mIU/mL	0.014	0.2
hGH	ng/mL	0.0006	0.002
hLH	mIU/mL	0.000	0.2
Inhibin A	pg/mL	0.34	1
Insulin	μIU/mL	0.000	0.03
Myoglobin	ng/mL	0.16	1
NT‐proBNP	ng/L	1.2	10
CA125	U/mL	0.00	0.5
p2PSA	pg/mL	0.361	3
PCT	ng/mL	0.0018	0.01
Progesterone	ng/mL	0.020	0.1
Prolactin	ng/mL	0.008	0.25
PSA	ng/mL	0.0000	0.008
PTH	pg/mL	0.02	1
Estradiol	pg/mL	2.391	15
Testosterone	ng/dL	0.000	0.1
Thyroglobulin	ng/mL	0.006	0.1
hsTnI	pg/mL	0.08	2.3
Total T3	ng/dL	7.0	10
TSH	μIU/mL	0.0000	0.005
uE3	ng/mL	0.0000	0.017
Vitamin B12	pg/mL	8.9	50

*Note:* All analytes demonstrated LoB values meeting acceptance criteria relative to manufacturer claims.

Abbreviations: AFP, alpha‐fetoprotein; AMH, anti‐Müllerian hormone; CA 125, cancer antigen 125; CA 15‐3, cancer antigen 15‐3; CA 19‐9, cancer antigen 19‐9; CEA, carcinoembryonic antigen; CK‐MB, creatine kinase‐MB; DHEA‐S, dehydroepiandrosterone sulfate; Free PSA, free prostate‐specific antigen; Free T3, free triiodothyronine; Free T4, free thyroxine; hFSH, human follicle‐stimulating hormone; hGH, human growth hormone; hLH, human luteinizing hormone; hsTnI, high‐sensitivity troponin I; NT‐proBNP, N‐terminal pro‐B‐type natriuretic peptide; p2PSA, [−2] pro‐prostate‐specific antigen; PCT, procalcitonin; PSA, prostate‐specific antigen; PTH, parathyroid hormone; Total T3, total triiodothyronine; TSH, thyroid‐stimulating hormone; uE3, unconjugated estriol; β‐hCG, beta‐human chorionic gonadotropin.

### 3.4 Method Comparison

Method comparison results are summarized in Table 4. Deming regression analyses demonstrated strong analytical agreement between the DxI 9000 and comparator platforms, including the DxI 800, Dimension EXL 200, and VIDAS 3 systems. Across all evaluated analytes, *R* ^2^ exceeded 0.95, and observed biases were within 0.8 × TEa.

**TABLE 4:** Deming regression results comparing the DxI 9000 to the DxI 800, Dimension, and VIDAS 3 systems across 36 analytes.

Analyte	Unit	Comparator method	*N*	Slope	Intercept	*R^2^*	Bias (%)
AFP	ng/mL	DxI 800	41	0.988	6.98	0.9997	5.12
AMH	ng/mL	DxI 800	47	0.942	0.06	0.9972	−4.17
CA15‐3	U/mL	DxI 800	48	0.959	0.85	0.9989	−0.94
CEA	ng/mL	DxI 800	46	1.033	−0.42	0.9998	1.16
CK‐MB	ng/mL	DxI 800	32	1.051	−0.27	0.9976	4.40
Cortisol	μg/dL	DxI 800	42	0.983	0.56	0.9939	2.69
DHEA‐S	μg/dL	DxI 800	70	1.010	−4.63	0.9923	−1.17
Ferritin	ng/mL	DxI 800	41	1.006	5.84	0.9989	3.73
Folate	ng/mL	DxI 800	48	0.962	0.15	0.9927	−1.18
Free PSA	ng/mL	DxI 800	40	1.025	0.01	0.9964	3.34
Free T3	pg/mL	DxI 800	41	1.013	0.18	0.9916	5.72
Free T4	ng/dL	DxI 800	44	0.945	0.09	0.9973	−0.40
CA19‐9	U/mL	DxI 800	40	0.915	0.08	0.9958	−7.75
β‐hCG	mIU/mL	DxI 800	45	1.028	0.17	0.9997	2.95
hFSH	mIU/mL	DxI 800	47	1.005	0.33	0.9979	1.96
hGH	ng/mL	DxI 800	43	1.040	0.25	0.9966	8.18
hLH	mIU/mL	DxI 800	44	1.144	−0.37	0.9978	10.73
Inhibin A	pg/mL	DxI 800	43	0.991	0.39	0.9979	1.39
Insulin	μIU/mL	DxI 800	42	1.096	−0.12	0.9969	9.03
Myoglobin	ng/mL	Dimension	43	0.860	−2.94	0.9991	−14.66
NT‐proBNP	ng/L	VIDAS 3	35	0.845	215.5	0.9914	−10.60
CA125	U/mL	DxI 800	39	0.960	8.35	0.9993	−0.91
p2PSA	pg/mL	DxI 800	38	1.179	−0.35	0.9936	12.81
PCT	ng/mL	DxI 800	42	1.067	−0.01	0.9980	6.49
Progesterone	ng/mL	DxI 800	35	0.931	−0.06	0.9892	−7.83
Prolactin	ng/mL	DxI 800	43	1.137	−0.34	0.9993	11.32
PSA	ng/mL	DxI 800	41	1.033	−0.10	0.9982	2.06
PTH	pg/mL	DxI 800	44	1.037	2.45	0.9966	5.79
Estradiol	pg/mL	DxI 800	44	0.862	13.08	0.9978	−9.99
Testosterone	ng/dL	DxI 800	41	0.946	0.06	0.9938	−3.46
Thyroglobulin	ng/mL	DxI 800	29	1.057	−1.20	0.9991	3.50
hsTnI	pg/mL	DxI 800	41	1.043	−0.03	0.9998	1.31
Total T3	ng/dL	DxI 800	43	0.839	0.18	0.9881	−2.12
TSH	μIU/mL	DxI 800	44	0.990	0.17	0.9983	1.17
uE3	ng/mL	DxI 800	30	0.972	0.04	0.9955	−4.72
Vitamin B12	pg/mL	DxI 800	44	1.097	5.6	0.9906	10.90

Abbreviations: AFP, alpha‐fetoprotein; AMH, anti‐Müllerian hormone; CA125, cancer antigen 125; CA15‐3, cancer antigen 15‐3; CA19‐9, cancer antigen 19‐9; CEA, carcinoembryonic antigen; CK‐MB, creatine kinase‐MB; DHEA‐S, dehydroepiandrosterone sulfate; Free PSA, free prostate‐specific antigen; Free T3, free triiodothyronine; Free T4, free thyroxine; hFSH, human follicle‐stimulating hormone; hGH, human growth hormone; hLH, human luteinizing hormone; hsTnI, high‐sensitivity troponin I; NT‐proBNP, N‐terminal pro‐B‐type natriuretic peptide; p2PSA, [−2] pro‐prostate‐specific antigen; PCT, procalcitonin; PSA, prostate‐specific antigen; PTH, parathyroid hormone; Total T3, total triiodothyronine; TSH, thyroid‐stimulating hormone; uE3, unconjugated estriol; β‐hCG, beta‐human chorionic gonadotropin.

For most analytes, approximately 40 patient specimens covering a broad analytical measurement range were included in the comparison analysis. For seven analytes—CK‐MB, progesterone, thyroglobulin, unconjugated estriol (uE3), [−2] pro‐prostate‐specific antigen (p2PSA), N‐terminal pro‐B‐type natriuretic peptide (NT‐proBNP), and CA 125—the number of available specimens was below 40 because of limited specimen availability during the study period.

**FIGURE 1:**
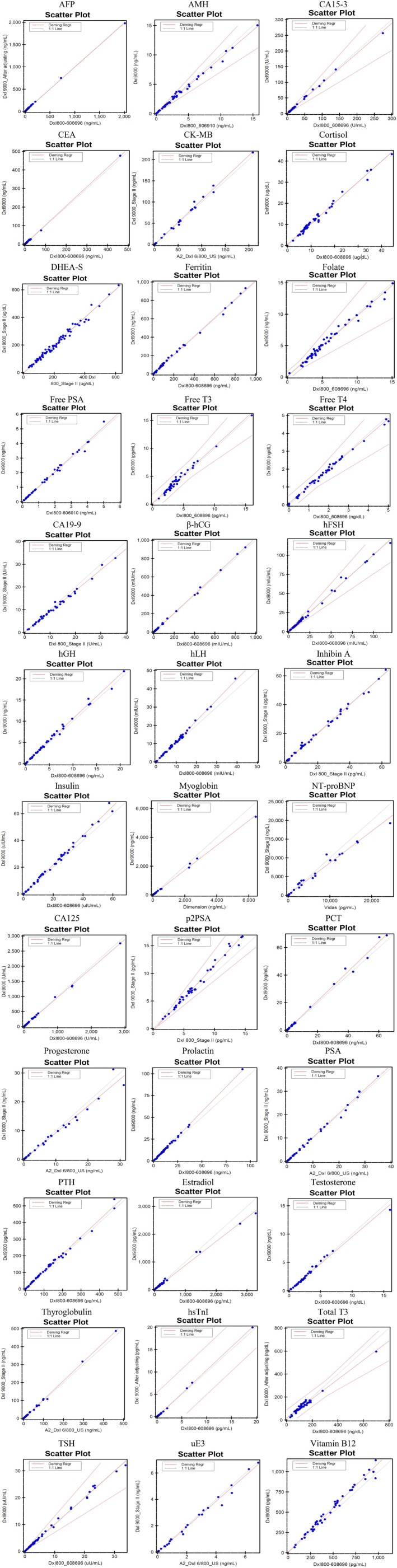
Representative Deming regression scatter plots showing method comparison between the DxI 9000 Access Immunoassay Analyzer and comparator platforms (DxI 800, Dimension EXL 200, and VIDAS 3) across selected analytes. The plots demonstrate strong correlation between systems across clinically relevant concentration ranges.

Representative Deming regression scatter plots illustrating the relationship between the DxI 9000 and comparator platforms are shown in Figure 1, demonstrating a strong correlation across the evaluated analytical ranges.

For estradiol, comparison results are presented with consideration of assay configuration differences, as the DxI 800 employs a conventional estradiol assay, whereas the DxI 9000 utilizes a sensitive estradiol formulation.

### 3.5 Carryover

Carryover results are summarized in Table 5. For the evaluated analytes, calculated carryover values were below the predefined acceptance criterion of three times the standard deviation of the Low–Low replicates.

**TABLE 5:** Carryover performance assessment using alternating high–low sample sequences.

Analyte	*L* (mean)	*H* (mean)	Carryover	Acceptance criterion^a^
β‐hCG (mIU/mL)	3.044	1308	0.032	0.215
hsTnI (pg/mL)	0.00380	500.067	0.00022	0.00082

Abbreviations: hsTnI, high‐sensitivity troponin I; β‐hCG, beta‐human chorionic gonadotropin.

^a^ Acceptance criterion defined as ≤ 3 × SD of Low–Low replicates, based on the EP Evaluator Carryover Report Interpretation Guide.

### 3.6 Reference Interval Verification

Reference interval verification results are summarized in Table 6. Verification was completed for 29 analytes, all of which met the predefined acceptance criteria, with at least 18 of 20 results (≥ 90%) falling within the corresponding manufacturer‐defined reference intervals. Verification of estradiol and prolactin was performed using male specimens only. Assays based on clinical decision thresholds rather than population‐based reference intervals, including p2PSA and Free PSA, were not included in the verification analysis.

**TABLE 6:** Reference interval verification results for 29 analytes on the DxI 9000 Access Immunoassay System using 20 healthy individuals.

Analyte	Unit	Reference interval
AFP	ng/mL	0–9
AMH	ng/mL	≥ 1
CA15‐3	U/mL	0–23.5
CEA	ng/mL	0–5
CK‐MB	ng/mL	0.6–6.3
Ferritin (Female)	ng/mL	11–306.8
Ferritin (Male)	ng/mL	23.9–336.2
Folate	ng/mL	≥ 4
Free T3	pg/mL	2–4
Free T4	ng/dL	0.54–1.4
CA19‐9	U/mL	0–35
β‐hCG	mIU/mL	0–5
hFSH (Male)	mIU/mL	1.3–19.3
hGH (Female)	ng/mL	0–3.6
hGH (Male)	ng/mL	0–1
hLH (Male)	mIU/mL	1.2–8.6
Insulin	μIU/mL	1.9–23
Myoglobin (Female)	ng/mL	14.3–65.8
Myoglobin (Male)	ng/mL	17.4–105.7
NT‐proBNP	ng/L	0–125
CA125	U/mL	0–35
PCT	ng/mL	0–0.5
Progesterone (Male)	ng/mL	0.14–2.06
Prolactin (Male)	ng/mL	2.6–17.0
PSA	ng/mL	0–4
PTH	pg/mL	15–88
Estradiol (Male)	pg/mL	0–33.1
Testosterone (Female)	ng/dL	0.1–0.8
Testosterone (Male)	ng/dL	2.78–7.64
Thyroglobulin	ng/mL	0–55
hsTnI	pg/mL	0–17.5
Total T3	ng/dL	70–170
TSH	μIU/mL	0.38–5.33
Vitamin B12	pg/mL	180–914

*Note:* All analytes met acceptance criteria, with ≥ 90% of results falling within the manufacturer‐provided reference intervals.

Abbreviations: AFP, alpha‐fetoprotein; AMH, anti‐Müllerian hormone; CA125, cancer antigen 125; CA15‐3, cancer antigen 15‐3; CA19‐9, cancer antigen 19‐9; CEA, carcinoembryonic antigen; CK‐MB, creatine kinase‐MB; DHEA‐S, dehydroepiandrosterone sulfate; Free PSA, free prostate‐specific antigen; Free T3, free triiodothyronine; Free T4, free thyroxine; hFSH, human follicle‐stimulating hormone; hGH, human growth hormone; hLH, human luteinizing hormone; hsTnI, high‐sensitivity troponin I; NT‐proBNP, N‐terminal pro‐B‐type natriuretic peptide; p2PSA, [−2] pro‐prostate‐specific antigen; PCT, procalcitonin; PTH, parathyroid hormone; Total T3, Total triiodothyronine; TSH, thyroid‐stimulating hormone; β‐hCG, beta‐human chorionic gonadotropin.

### 3.7 Additional Functional Evaluations

Additional functional evaluation results are summarized as follows. Inter‐pipettor consistency testing across four pipettors yielded percent bias values ranging from 0.18% to 4.63%. Low‐volume precision testing performed in dilution mode resulted in coefficients of variation of 2.44% for AFP (4 μL) and 1.86% for β‐hCG (2 μL).

TTFR assessment using sequential STAT samples showed shorter elapsed times from sample loading to result availability compared with historical workflow data from the predecessor DxI 800 platform, with reductions of at least 5 min observed for hsTnI (17 → 11 min), β‐hCG (18 → 12 min), and PCT (20 → 13 min).

## 4 Discussion

This single‐center evaluation demonstrates that the DxI 9000 Access Immunoassay System provides analytically reliable performance across a broad test menu of 36 immunoassay analytes while supporting routine clinical laboratory operation. By integrating standardized analytical verification with selected functional assessments, this study offers an integrated characterization of system performance under real‐world laboratory conditions, extending prior platform‐specific evaluations that have focused on narrower assay subsets [1, 8].

With respect to analytical performance, precision results across the evaluated analytes were within manufacturer‐defined acceptance criteria and aligned with CLSI EP05‐A3 recommendations [9, 17]. Stable repeatability and within‐laboratory precision across multiple concentration levels and assay categories are essential for routine diagnostic testing, particularly in settings requiring longitudinal patient monitoring. The observed performance is consistent with previous multi‐analyte studies of contemporary chemiluminescent immunoassay platforms, which have emphasized the importance of reproducible analytical behavior across diverse diagnostic applications [1, 8].

Method comparison analyses further indicated a high degree of analytical agreement between the DxI 9000 and established comparator platforms, including the DxI 800, Dimension, and VIDAS 3 systems. Across all evaluated analytes, coefficients of determination exceeded 0.95 and observed biases remained within 0.8 × TEa [18, 21]. Such concordance suggests that results generated by the DxI 9000 are analytically comparable to those obtained from existing platforms, thereby supporting continuity of longitudinal result interpretation during system transitions in routine clinical practice.

Analytical sensitivity assessment suggests that LoB values met or exceeded manufacturer‐stated performance claims for all assays in accordance with CLSI EP17‐A2 methodology [12]. Adequate low‐end analytical sensitivity is particularly relevant for assays used in early disease detection and serial monitoring, including cardiac, oncologic, and endocrine biomarkers, where small changes in analyte concentration may carry clinical significance. The observed sensitivity performance is consistent with recent immunoassay studies reporting improved signal discrimination at low analyte concentrations [18, 19].

Beyond core analytical performance, selected functional evaluations provided insight into the operational implications of system implementation in acute‐care settings. The observed reductions in TTFR for STAT assays, relative to historical workflow data from the predecessor platform, indicate improved analytical throughput under high‐acuity testing conditions. Timely reporting of high‐sensitivity cardiac biomarkers is a critical component of emergency department workflows, and prior studies have demonstrated that accelerated troponin testing can shorten laboratory turnaround time and improve emergency department throughput when integrated into structured clinical pathways [22]. In addition, incorporation of high‐sensitivity troponin assays into accelerated decision‐making algorithms, such as the HEART Pathway, has been associated with increased early discharge rates, reduced unnecessary hospital admissions, and preserved patient safety [23]. Taking together, the TTFR improvements observed with the DxI 9000 suggest that gains in analytical efficiency may translate into broader operational benefits, including enhanced patient flow, more efficient resource utilization, and improved overall emergency department performance.

Implementation of the DxI 9000 was also associated with reduced routine maintenance requirements compared with the predecessor platform. Simplified maintenance procedures translated into measurable reductions in technologist labor time in routine operation. Although modest at the individual system level, such cumulative time savings may have practical implications for staff allocation and workload distribution in high‐throughput laboratories facing ongoing workforce constraints. These operational considerations complement analytical performance metrics and reflect the multifaceted factors influencing system suitability in contemporary clinical laboratories.

Several limitations of this study should be acknowledged. First, the method comparison analysis did not reach the CLSI EP09‐A3 recommended sample size of at least 40 patient specimens for all analytes. Although most assays were evaluated using approximately 40 samples, seven analytes—CK‐MB, progesterone, thyroglobulin, unconjugated estriol (uE3), [−2] pro‐prostate‐specific antigen (p2PSA), N‐terminal pro‐B‐type natriuretic peptide (NT‐proBNP), and CA 125—were assessed using fewer specimens because of limited specimen availability and relatively lower routine testing frequency during the study period. Efforts were made to include specimens spanning the widest possible analytical measurement range encountered in routine clinical practice. Nevertheless, the comparison results for these analytes should be interpreted as implementation‐phase verification data rather than fully powered CLSI EP09‐A3 validation datasets.

Second, this evaluation was conducted at a single institution and included a selected subset of the available assay menu; multicenter studies would provide broader insight into system performance across diverse patient populations and laboratory workflows. Third, reference interval verification was limited to selected adult cohorts and may not fully represent pediatric, geriatric, or physiologically complex populations. Finally, accuracy assessment for certain analytes relied on manufacturer‐defined targets or external quality assessment data in contexts where peer‐group statistics were limited. Future investigations incorporating multicenter analytical comparisons, expanded workflow assessments, and outcome‐based analyses linking analytical performance to clinical endpoints would further clarify the clinical and operational impact of high‐throughput immunoassay platforms.

## 5 Conclusion

This study provides a comprehensive single‐center evaluation of the analytical and functional performance of a high‐throughput chemiluminescent immunoassay system across a broad test menu of 36 analytes. The findings demonstrate that the DxI 9000 achieves reliable analytical performance, including acceptable precision, accuracy, analytical sensitivity, and method comparability, in accordance with established verification standards. In addition, selected functional assessments indicate improved workflow efficiency under routine and STAT testing conditions.

Together, these results support the suitability of the DxI 9000 for routine use in tertiary‐care clinical laboratories requiring both analytical robustness and efficient operational performance. While further multicenter and outcome‐based studies are warranted, the present evaluation contributes practical evidence regarding system performance under real‐world laboratory conditions.

## Author Contributions

**Chiung‐Tzu Hsiao** and **Chao‐Ching Liao:** supervision, investigation, writing – original draft. **Yu‐Chang Chang**, **Hui‐Ju Chang**, and **Tz‐Ya Huang:** investigation, review and editing. **Jen‐Shiou Lin** and **Po‐Ren Hsueh:** supervision, investigation, review and editing. All authors accept responsibility for the entire content of this manuscript and have approved its submission.

## Funding

The authors have nothing to report.

## Ethics Statement

This study was reviewed and approved in accordance with the institutional Test Development and Assessment Management Procedure at China Medical University Hospital. The project was registered under approval number CMUH‐QP‐0504‐001‐2301.

## Consent

The requirement for informed consent was waived for all individuals included in this study.

## Conflicts of Interest

The authors declare no conflicts of interest.

## Data Availability

The data that support the findings of this study are available from the corresponding author upon reasonable request.
